# Supplementary material for: Efficient encoding of spectrotemporal information for bat echolocation
Source: PLoS Comput Biol. 2021 Jun 28;17(6):e1009052. doi: 10.1371/journal.pcbi.1009052 (PMC8270447; doi:10.1371/journal.pcbi.1009052)
Supplement: S1 Text — Fig A. Results from the procedure used to determine a realistic level of internal noise also used in references [7, 11, 27]. As described in the main text, for each level of noise σ we determined P[∑C6+Nσ>∑C0+Nσ], using a Monte Carlo approach. This graph gives the probability P[⋅] as a function of noise level σ. The value of σ at which P[⋅] = 0.75 was taken as the noise level throughout this paper. Fig B. Ensonification device. The custom-built device consisted of two Knowles microphones embedded in a 3D printed housing and a Sensecomp 7000 emitter. The device was mounted on a tripod. Fig C. Average cochleogram, derived from the ensonification data. b Binarized average cochleogram showing where samples are at least 20% of the maximum value. Fig D. (Left) Distribution of the values of the cochleograms collected in this paper. (Right) Distribution of the 25 filter output values for all ensonification data echoes used in the this paper. As the value of the entropy H(S) depends on the quantization used, we encoded all samples as doubles for this calculation. (PDF) [file pcbi.1009052.s001.pdf]

S1 Text

Center frequencies of the Gammatone Filterbank

We used an implementation of Wiegrebe's [1] functional model of inner ear processing. As part of this, echo signals were passed through a gammatone filterbank. The center frequencies of the 20 filters were spaced by the Equivalent Rectangular Bandwidths [2]. The center frequencies used were the following (here, rounded to the nearest Hertz): $f_c =$[16160, 18028, 20109, 22427, 25009, 27886, 31090, 34660, 38636, 43066, 48000, 53497, 59620, 66441, 74040, 82504, 91934, 102438, 114139, 127174].

Noise model

Formally, with $\mathbf{S_{ref}}$ denoting the cochleogram corresponding with the rewarded stimulus (=reference echo amplitude) and $\mathbf{S_{+5}}$ the cochleogram corresponding with the unrewarded stimulus (echo amplitude 5 dB higher than the reference amplitude), we determined the value of σ that satisfies the equation,

$$\begin{aligned} P[\sum_{i,j}(S_{+5}(t_i,f_j) + \mathcal{N}(0,\sigma) - S_{ref}(t_i,f_j))^2 \\ > \\ \sum_{i,j}(S_{ref}(t_i,f_j) + \mathcal{N}(0,\sigma) - S_{ref}(t_i,f_j))^2] \\ = 0.70 \end{aligned} \tag{1}$$

with i, j indices running over the time-frequency bins of the cochleograms. The result of this procedure is shown in figure A.

To derive this decision rule we model the 2AFC experiment reported on by Heinrich et al. [5] by writing the stimulus received from the right speaker as $\mathbf{S_R} = \mathbf{S_{ref}} + \boldsymbol{\delta}_R$ and the stimulus received from the left speaker as $\mathbf{S_L} = \mathbf{S_{+5}} + \boldsymbol{\delta}_L$, with $\boldsymbol{\delta}_L, \boldsymbol{\delta}_R$ denoting the internal noise on the received left and right speaker stimuli respectively, $\mathbf{S_{ref}}$ the rewarded stimulus (faintest echo) and $\mathbf{S_{+5}}$ the louder (unrewarded) stimulus. Note that we will assume that the right speaker emits the rewarded stimulus. Assuming the bat has no information about the loudness of the unrewarded stimulus it has to base its decision on what loudspeaker emits the sound that resembles the rewarded stimulus the most. Hence, a correct decision is reached if the bat judges the likelihood of its internal representation of the received stimulus S_R corresponding with the rewarded stimulus S_{ref} to be larger than that for the received stimulus S_L, or formally

$$p(\| \mathbf{S_L} - \mathbf{S_{ref}} \| = 0) < p(\| \mathbf{S_R} - \mathbf{S_{ref}} \| = 0). \tag{2}$$

In terms of the model of the internal noise as independent and identically distributed zero mean Gaussian noise, this decision rule can be written as

$$\frac{e^{-0.5\|\mathbf{S_R}-\mathbf{S_{ref}}\|^2/\sigma^2}}{e^{-0.5\|\mathbf{S_L}-\mathbf{S_{ref}}\|^2/\sigma^2}} > 1. \tag{3}$$

Taking logarithms and dropping common factors this is equivalent to the decision rule

$$\| \mathbf{S_R} - \mathbf{S_{ref}} \|^2 - \| \mathbf{S_L} - \mathbf{S_{ref}} \|^2 < 0 \tag{4}$$

which corresponds with the rule used in Eq. 1.

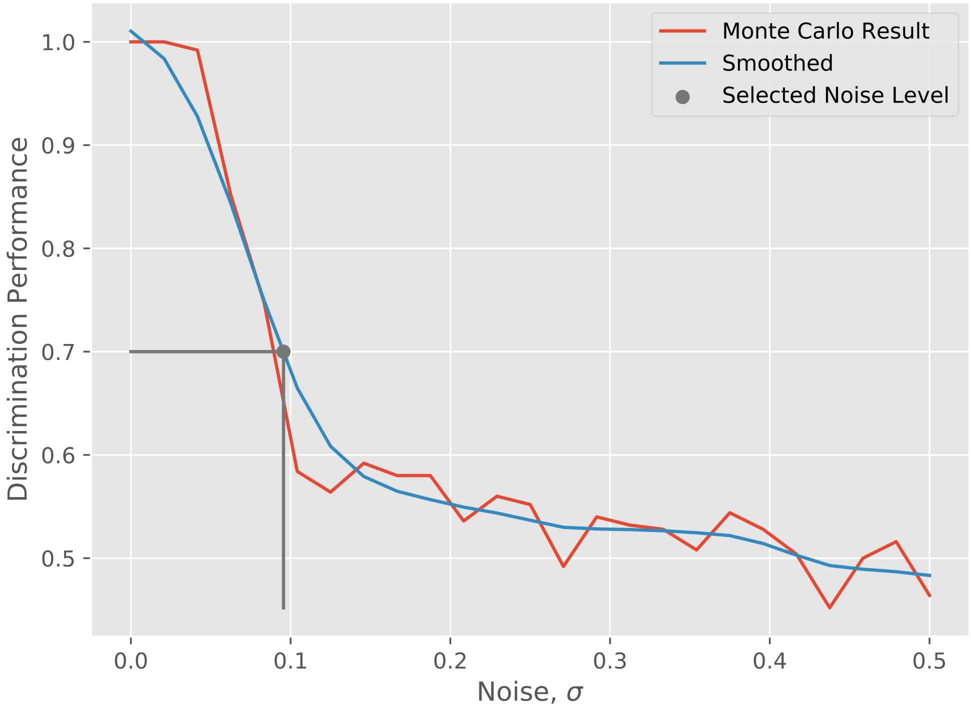

Fig A. Results from the procedure used to determine a realistic level of internal noise also used in references [1,3,4]. As described in the main text, for each level of noise σ we determined $P\left[\sum C_6 + \mathcal{N}_\sigma > \sum C_0 + \mathcal{N}_\sigma\right]$, using a Monte Carlo approach. This graph gives the probability $P[\cdot]$ as a function of noise level σ. The value of σ at which $P[\cdot] = 0.75$ was taken as the noise level throughout this paper.

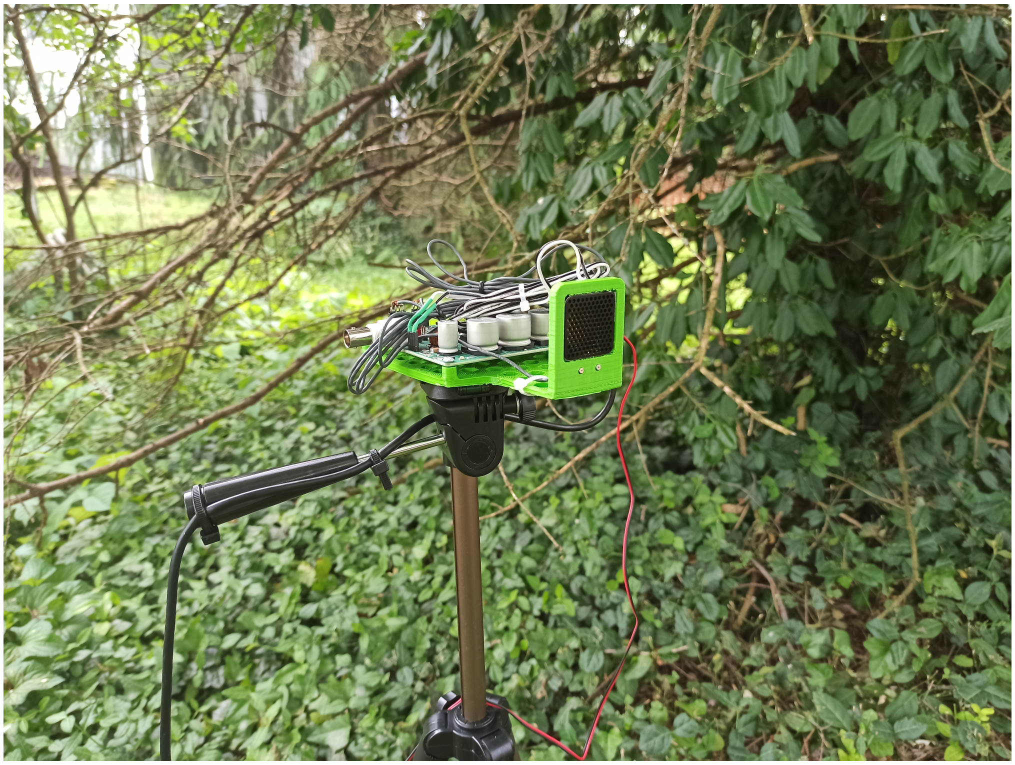

Fig B. Ensonification device. The custom-built device consisted of two Knowles microphones embedded in a 3D printed housing and a Sensecomp 7000 emitter. The device was mounted on a tripod.

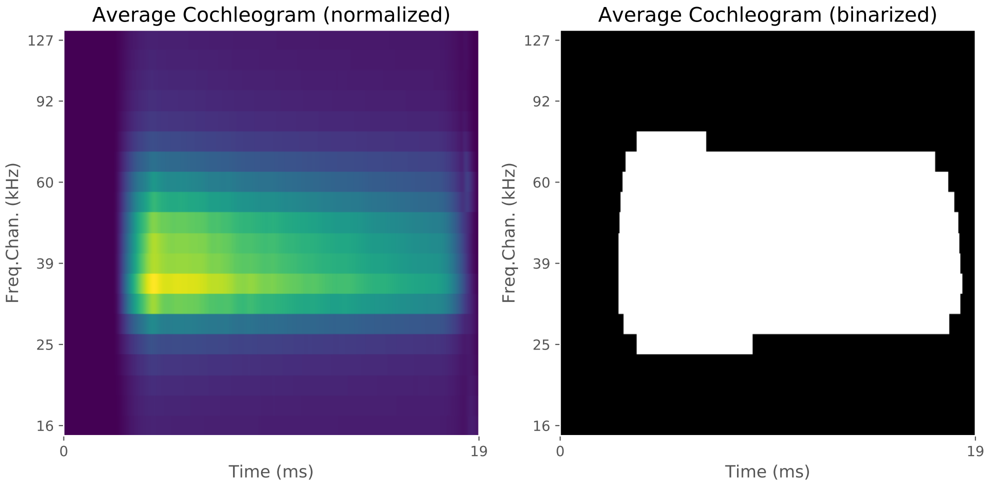

Fig C. **a** Average cochleogram, derived from the ensonification data. **b** Binarized average cochleogram showing where samples are at least 20% of the maximum value.

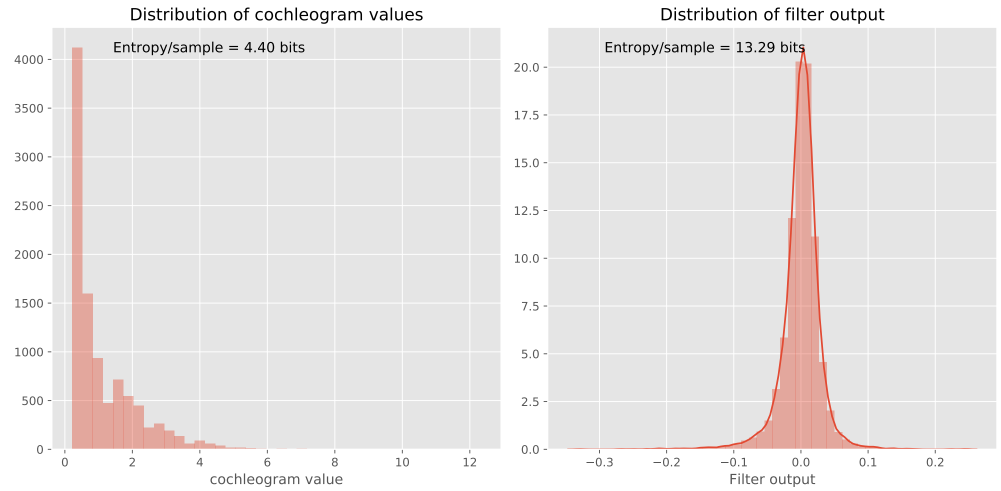

Fig D. (Left) Distribution of the values of the cochleograms collected in this paper. (Right) Distribution of the 25 filter output values for all ensonification data echoes used in the this paper. As the value of the entropy $H(S)$ depends on the quantization used, we encoded all samples as doubles for this calculation.

References

1. Wiegrebe L. An autocorrelation model of bat sonar. Biological cybernetics. 2008;98(6):587–595.
2. Moore BC, Glasberg BR. Suggested formulae for calculating auditory-filter bandwidths and excitation patterns. The journal of the acoustical society of America. 1983;74(3):750–753.
3. Dau T, Püschel D, Kohlrausch A. A quantitative model of the “effective”signal processing in the auditory system. I. Model structure. The Journal of the Acoustical Society of America. 1996;99(6):3615–3622.
4. Vanderelst D, Steckel J, Boen A, Peremans H, Holderied MW. Place recognition using batlike sonar. Elife. 2016;5:e14188.
5. Heinrich M, Warmbold A, Hoffmann S, Firzlaff U, Wiegrebe L. The sonar aperture and its neural representation in bats. Journal of Neuroscience. 2011;31(43):15618–15627.
