# Supplementary material for: Efficient encoding of spectrotemporal information for bat echolocation
Source: PLoS Comput Biol. 2021 Jun 28;17(6):e1009052. doi: 10.1371/journal.pcbi.1009052 (PMC8270447; doi:10.1371/journal.pcbi.1009052)
Supplement: Attachment — Submitted filename: reply to reviewers plos comp bio.pdf [file pcbi.1009052.s002.pdf]

Reviewer 1

Comment: One criticism I have is that the authors could use the neural representation of the inner ear which already reduces the high dimension correlogram to a lower dimension in a way that is more biological than an ICA analysis. I know of no evidence that the brain transforms sensory information into independent axes – in fact this was the view in the vision literature ca. 2 centuries ago, but is not generally accepted today. This criticism is related to the authors statement that: “The main difference between this study and these previous studies is that the set of filters used in the proposed compressive encoding is not custom-built for one particular task but is derived from a database of ecologically valid echo signals.” From my understanding, the authors used ICA which has some compressive definitions, but one could say the same thing about PCA which has been used before. I thus do not understand why this statement is correct.

Reply: It is true that PCA has been used before (e.g. Yovel et al., 2008; Wotton & Jenison, 1997) to compress sonar echoes. However, it is not so much the specific compression procedure used nor the fact that specific echo data can be compressed for a specific task, that we believe is the most important point raised in the current paper. The true contribution, we believe, lies in the realization that, different from previous work we are aware of, the basic components, i.e. the encoded features, used in the compression can be learned from non-specific echo data collected in settings that are not related to those of the experiments we modelled. Concretely, the basic components used for the ICA are derived from a series of echo snapshots collected from different natural environments the sonar was operating in. From the experiments described in the Yovel et al., 2008 paper for example, one can conclude that the echoes from five different plant species can be compressed to 250 numbers without losing the essential information required to distinguish between these five classes of echoes. But, from the current paper, we argue, a more general conclusion can be drawn, i.e. echo signals can be compressed into a small set of numbers (25 in the modelled experiments), learned from a set of echoes collected through daily use of the sonar system, while maintaining the ability to perform a broad range of fundamental echolocation tasks. We have edited the abstract and the significance statement to stress this point.

Comment: My second main major concern is the use of neural networks and their tendency to overfit the data, even when training and testing sets are supposedly separated. For example, in many studies samples are divided randomly between training and testing sets, even though samples are often highly correlated, e.g., when two echoes are collected from nearly the same angle – they will be very similar. In such cases, there is essentially no separation between the training and the testing sets. From reading the manuscript, it seems to me that a similar approach was taken here. If this is correct, the performance of the network shows an ability to learn a set of examples rather than to generalize. To test this, or prove it wrong, the authors should measure similarity between examples in the training and testing sets and show that there are no or few examples which are very similar.

Reply: We agree with the reviewer that there is a difference between a neural network memorizing the input data and one generalizing from the input data. However, in this particular instance, the neural networks were only used as a means to show that no essential information is lost through the compressive encoding of the sonar echoes. Any technique that can be employed to map the compressed encodings of the echoes onto correct experimental responses could be used instead. Hence, whether the neural network memorizes its input data or generalizes from them to generate its response has no bearing on the information-carrying capacity of the compressed encodings. Indeed, in either case, success at the task would show that the required information is still present in the network’s input, whereas failure would show that information was lost.

Minor comments

Comment: L119 – The authors write: "We averaged across these three repeats to increase the signal to noise ratio". Was the averaging done at the correlogram level? Averaging at the level of the time-signal would change the signal dramatically

Reply: We averaged at the cochleogram level. We have edited the text to make this clearer.

Comment: I think that the notation of the math (starting around line 135) is confusing. In theory, the ICA could have run directly on the correlograms, but from what I understood the authors first use PCA to reduce correlogram dimensionality to 25 and only then ran an ICA analysis. The application of a PCA in the middle is not shown in figure 1, so it is not fully clear to me if and when it was performed.

Reply: Yes, a PCA was performed, but as it is seen as a preprocessing step contained within the ICA analysis by the software we use, we consider it an integral part of the ICA step shown in Fig. 1 and do not show it separately. We have clarified this in the text. We have also simplified the description somewhat with more focus on the reasoning and less on the formal model. We have added a reference (Hyvarinen, 2000) for those readers that are interested in a more detailed formal description of the technique.

Comment: Moreover, the authors use S (capital or not) to notate three different things. I think this could improve much.

Reply: We noticed that there was indeed some confusion with the notation, different symbols are now used with S referring to spectrograms only.

Comment: Note that Yovel et al. 2008 already showed that a much lower dimension representation of the echo can be used to perform object classification. If I am not mistaken, they used PCA to reduce the data to ca. 200 dimensions, essentially using a similar approach to that described here.

Reply: Indeed, in the paper by Yovel et al. 2008 a PCA (250 components) is used as a preprocessing stage before classification of the plant echo spectrograms by an SVM is attempted. First, it should be noted that the compression introduced in this manuscript is an order of magnitude higher (25 versus 250 components), resulting in a significant further reduction of the amount of information that needs to be processed. However, as we did not model plant classification, we make no claims as to how successful our compression would be in that task. But regardless of the exact compression factor, we believe that the most important novelty of our approach is that, as stated above, the encoded features used in the compression are learned from non-specific echo data collected in settings that are unrelated to the modeled tasks. Indeed, the components derived by Yovel et al. 2008 were derived from plant echoes, with the exclusive goal of classifying plant echoes. The following is a quotation from that paper, making this point:

In every experiment, the eigenvectors were calculated for the covariance matrix of the training set exclusively. The PCA transformation reduced the dimensionality of the data so that each echo could now be represented by a 250-dimensional vector.

Comment: Figures are not numbered according to their appearance in the paper

Reply: We could find no mismatch between the order of references and appearances of the figures in the text. Perhaps, this is something that could be clarified by the editorial office at a later stage.

Reviewer 2

Comment: The first citation in the paper is [55]. What's happening is that references are listed alphabetically in the Reference list first, and only then referenced in the main text. This can be confusing at times for the reader who is trying to check the citations.

Reply: We have changed the order of the references.

Comment: Line 17-18: the sentence is unclear. Probably related to the use of the word "foremost" in this context? For example, do the authors mean that the timing of echo arrival is the most relevant information?

Reply: Sentences are rephrased.

Comment: Line 29: "Pose" recognition by Vanderelst et al [52]?

Reply: Sentences are rephrased.

Comment: Line 105: "just over 1000 echoes". Is it above 1000 echoes, as this suggests, or were there N=1000 echoes, as stated above (l. 70)?

Reply: The exact number of echoes was 1014. We have updated the text accordingly.

Comment: Line 108: This part is unclear to me. Echoes were 23 ms long. Were all echoes equally long, regardless on the reflector (21 locations, etc.), or was echo length defined as 23 ms for convenience?

Reply: The maximum echo length was chosen based on the available onboard memory of the sonar data acquisition board used and all echoes had the same (maximum) length. We have added this to the paper.

Comment: It is also unclear how the maximal range is estimated from the echo duration; if this is derived by the authors, then it should be explained in the Methods. If it's based on previous literature (e.g. [49]), it should be specified.

Reply: The maximum range corresponding with the chosen echo duration is calculated as the distance to the reflector and back as covered by the reflected sound waves traveling at a speed of sound of 343m/sec. This was added to the text.

Comment: Line 127: hyperbolic FM sweeps are maximally Doppler-shift resilient. Can the authors provide a citation for this?

The following references were added:

- R. Altes, Sonar for generalized target description and its similarity to animal echolocation systems, JASA, Vol.59, No.1, January 1976.
- Holderied M.W., Baker C.J., Vespe M., Jones G., Understanding signal design during the pursuit of aerial insects by echolocating bats: tools and applications. Integr. & Comp. Biol. 2008 Jul; 48(1):74-84. doi: 10.1093/icb/icn035. Epub 2008 May 14.

Comment: As far as I understand, the de-chirping requires to know a reference signal (the way is done, for example for radar detection). The authors claim that the "bat has knowledge of its emission", yet no reference is provided for this, nor a mechanism suggested (e.g. efference copies?). Other models that

compensate the FM effect (like that of Wiegrebe, 2008) base this compensation on computing autocorrelations. Autocorrelations could be calculated from neural activity given, for example, a plausible architecture such as described by Shamma (2001, Trends in Cognitive Sciences). Such architecture requires no knowledge of a “reference” signal, like the de-chirping does. Why was the de-chirping implemented in the model? Would the FM compensation via autocorrelation produce the same results? Could the authors substantiate the phrase “the bat has knowledge of its emission” with a speculation of how this “knowledge” occurs in the bat’s brain?

Reply: We don’t know what mechanism is actually used by bats to take into account the knowledge of its own emissions, efference copies is an obvious possibility but the two possible mechanisms mentioned in the text are both biologically plausible mechanisms that do not rely on an explicit ‘reference signal’. In addition to the autocorrelation mechanism proposed in Wiegrebe 2008, there is also the SCAT model (Saillant et al., 1993), that uses the spike patterns in the auditory nerve generated by reception of the bat’s own vocalisation as an implicit ‘reference signal’ to dechirp the received echo signal through a series of delay lines and coincidence detectors, one for each cochlear frequency channel. As our ‘dechirping’ operation is, apart from a frequency-dependent gain, essentially an autocorrelation, we expect the results to be similar. The most important reason for us to introduce the dechirping operation is that it makes the derived components more independent from changes in the spectrotemporal shape of the call used by the bat. As many bats adapt their call depending on the circumstances, e.g. phase of hunting or clutter/interference conditions, a mechanism that removes these changes from the memorized compression components has obvious advantages.

Comment: Line 208: JND is not defined as an abbreviation.

Reply: Corrected

Line 221: where instead of were.

Reply: Corrected

Comment: Fig. 3e-h are mentioned first in the main text. Why not swap panels a-d (top) with e-h (bottom)? This way, the order would be consistent in the main text, and also individual component examples would be shown first, before exploring details about components in general.

Reply: figure was changed as suggested.

Comment: Fig. 3a: x-axis and label missing?

Reply: We added an x-axis for this panel (note, in the updated figure, this is now panel [e]).

Comment: Fig. 3d: although distance and time and linearly dependent, this relationship is not explicit in the manuscript. The authors write in the main text (l. 360) of “best delay”, when in the figure is shown “Filter best distance (m)”. I suggest to either make the relationship [ms] -> [m] explicit, or modify Fig. 3d to show best delays and not best distance for consistency.

Reply: The relationship between time and distance has been made explicit in the caption of Fig 3.

Comment: Lines 386-387: JND is not defined in the text, it would be better to define it and to specify the parameter you are referring to, i.e. sometimes JND is in ms, sometimes in dB.

Reply: JND has been defined in the text and we have specified for each reference whether the JND refers to echo delay or echo strength.

Comment: As enjoyable as the Information processing implications subsection is, I have my reserves as to how it fits into the Discussion. Given its reliance on calculations and data (e.g. Figs. S3, S4), could it be better placed in the Results? It is, at any rate, unusual to bring up new data and figures for the first time in the Discussion section.

Reply: We agree with the reviewer that this is somewhat unusual. In response to this comment, we tried to split out the section referred to into a part that would go in the results and a part that would be included in the discussion. However, separated from the discussion of the information-theoretical advantages of the encoding, we found that the point of the calculations was not easy to grasp and we ended up duplicating much of the text in both the result and the discussion section. Therefore, we reverted back to including this only once in the discussion. We apologize for this idiosyncrasy.

Comment: Line 594-96: The authors say, based on ref. 20, that bat's neurons show broader (frequency) tuning when being selective to longer target distance. Yet, they also say that this feature is reflected in the paper, in which increasing response time occurs with increasing target distance (from Fig. 3e-h, I suppose). Unless I miss something, broader frequency tuning (as per ref. 20) is not necessarily equivalent to increasing response times, although both seem to correlate with large delays. Perhaps it would be useful to clarify this.

Reply: The sentence in line 594 should refer to **delay-tuning**, not frequency tuning. We would interpret the data shown in Fig. 5 in Hechavarria et al. 2013 as an increase in the width of delay-tuning curves with increasing characteristic delay. The same can be seen from Figure 3e-h of our manuscript, showing that delay-tuning gets broader with best distance. We rephrased these sentences to clarify the point we want to make.

Comment: Line 599: frequency selectivity does not appear from IC on but from the cochlea onwards and persists along the pathway.

Reply: The reviewer is clearly right in that frequency selectivity exist from the cochlear onward, but delay selectivity does not. However, at least from the IC on, delay AND frequency selectivity have been established in the ascending auditory pathway. Hence, we rephrased the sentence accordingly.

Comment: Lines 593-95: what centers would support decision-making during echolocation in bats? I think the literature is very conflicting about this. Would it involve cortex or only subcortical structures?

Reply: We would guess that the frontal-cortical areas are most important for decision making during echolocation in bats as is typically assumed for other tasks in other mammals. However, we feel that it is beyond the scope of the manuscript to deal with the question of decision-making in detail. Nevertheless, we added a few lines at the end of the discussion section concerning this issue and follow another suggestion of the reviewer (see the answer to next comment).

Comment: Can the 25 filters reported here suffice also for communication call coding in this species?

Reply: While this is a very interesting question, we feel we have, at this stage, not much of interest to say on this matter. One general comment maybe: while we can expect the features of the echo signals to somehow encode the same spatial features of the environment irrespective of the particular species of bat, this might no longer be true for the features of communication calls as they are less constrained and can

evolve along very different paths for different species. Hence, we can expect such an encoding to be less general and more species-specific than the encoding presented here.

Comment: The neurophysiology part of the discussion is very "feedforward" focused. Yet it is almost certain that feedback pathways play a role when the bat vocalizes. For example, feedback activity could change the way the 25 filters reported here behave in different contexts. This should be pointed out to prevent the naïve reader from getting the impression that everything is feedforward in the bat brain.

Reply: We thank the reviewer for this suggestion. We have added a few lines pointing towards a possible role of top-down feedback on filter properties. New References added in this extra piece of text:

Duncan, J. An adaptive coding model of neural function in prefrontal cortex. Nat. Rev. Neurosci. 2, 820–829 (2001).

Everling, S., Tinsley, C.J., Gaffan, D. & Duncan, J. Selective representation of task relevant objects and locations in the monkey prefrontal cortex. Eur. J. Neurosci. 23, 2197–2214 (2006).

Fritz et al., 2010. Adaptive, behaviorally gated, persistent encoding of task-relevant auditory information in ferret frontal cortex. Nature Neuroscience

Gold, J.I. & Shadlen, M.N. The neural basis of decision making. Annu. Rev. Neurosci. 30, 535–574 (2007).

Miller, E.K. & Cohen, J.D. An integrative theory of prefrontal cortex function. Annu. Rev. Neurosci. 24, 167–202 (2001).

Comment: Fig. S4 is missing y-axis labels.

Also Fig. S4 (right). The figure caption reads that the distribution shown here is for 15 filter output values. Does that mean that there were 15 values? Or that this is the distribution of values from 15 filters? If the latter is correct, and there were 25 filters from the ICA analysis, is this a typo?

Reply: This has been corrected.
